# Prediction of the Thermal Conductivity of Refrigerants by Computational Methods and Artificial Neural Network

**DOI:** 10.3389/fchem.2017.00099

**Published:** 2017-11-15

**Authors:** Forouzan Ghaderi, Amir H. Ghaderi, Noushin Ghaderi, Bijan Najafi

**Affiliations:** ^1^Faculty of Chemistry, University of Isfahan, Isfahan, Iran; ^2^Department of Cognitive Neuroscience, University of Tabriz, Tabriz, Iran; ^3^Faculty of Engineering, Shahrekord University, Shahrekord, Iran; ^4^Department of Chemistry, Isfahan University of Technology, Isfahan, Iran

**Keywords:** thermal conductivity, transport properties, refrigerant, RF theory, ANN

## Abstract

**Background:** The thermal conductivity of fluids can be calculated by several computational methods. However, these methods are reliable only at the confined levels of density, and there is no specific computational method for calculating thermal conductivity in the wide ranges of density.

**Methods:** In this paper, two methods, an Artificial Neural Network (ANN) approach and a computational method established upon the Rainwater-Friend theory, were used to predict the value of thermal conductivity in all ranges of density. The thermal conductivity of six refrigerants, R12, R14, R32, R115, R143, and R152 was predicted by these methods and the effectiveness of models was specified and compared.

**Results:** The results show that the computational method is a usable method for predicting thermal conductivity at low levels of density. However, the efficiency of this model is considerably reduced in the mid-range of density. It means that this model cannot be used at density levels which are higher than 6. On the other hand, the ANN approach is a reliable method for thermal conductivity prediction in all ranges of density. The best accuracy of ANN is achieved when the number of units is increased in the hidden layer.

**Conclusion:** The results of the computational method indicate that the regular dependence between thermal conductivity and density at higher densities is eliminated. It can develop a nonlinear problem. Therefore, analytical approaches are not able to predict thermal conductivity in wide ranges of density. Instead, a nonlinear approach such as, ANN is a valuable method for this purpose.

## Introduction

Fluids, containing gases and liquids, have a wide range of applications in daily life and play an important role in modern industrial processes. Since refrigerants are crucial in the refrigeration industry, we must have a scientific knowledge about their transport properties. Computational methods that allow for the prediction of transport properties serve as valuable tools due to their time efficiency when compared to the time it would take to conduct the work in a laboratory. The effect of microscopic motions and the interactions of molecules on the transport properties of gases are considered to form the basis of these methods.

The transport properties of refrigerants have been investigated in numerous studies. Kandlikar et al. (1975) predicted the transport properties of R_12_ and R_22_ (Kandlikar et al., 1975). Assael at al. performed the absolute thermal conductivity measurement of liquid refrigerants R_11_ and R_12_ at the temperature range of 250–340 K and pressures from saturation to 30 MPa (Assael et al., 1992). In another study, the thermal conductivity of R_134a_, R_152a_, and R_123_ was evaluated using the transient hot-wire method (Gross et al., 1992). The viscosity coefficient of R_152_ was investigated by Krauss et al. (1996). A practical representation for the transport coefficients of pure refrigerants R_32_, R_125_, R_134a_, and R_125_ + R_32_ mixtures was presented by Kiselev et al. and was valid in the vapor-liquid critical region (Kiselev et al., 1999). Also, the transport properties of R14 have been examined in some studies (Smith and Pace, 1969; Rubio et al., 1985).

In 1917, Sydney Chapman and David Enskog solved the accurate equation for the transport properties of gases that had been presented by Maxwell and Boltzmann in 1860–1870 (Levine, 1995). Chapman-Enskog relations for monatomic gases and mixtures of these gases are expressed through the use of inter-atomic potential and temperature. In this theory, each coefficient of transport properties in gases is related to the collision integral (Ω) that depends on the value of potential, the nature of the interaction, and the reduced temperature (T^*^). The basis of the calculation of (Ω) in this work was the expansion of corresponding states presented by Najafi et al. (1983).

The Chapman-Enskog theory relates the effect of local binary molecular collisions. Furthermore, the role of higher-order molecular collisions is considered in the regions of higher density in gases. The existence of the internal degree of freedom in polyatomic gases has a considerable influence on thermal conductivity (λ), and thus thermal conductivity cannot be definitely calculated by the Chapman-Enskog theory (Maitland et al., 1978). In 1980, Rainwater and Friend proposed a microscopic theoretical methodology for calculating the second transport virial coefficients of moderately dense gases (up to 2 mol.dm^−3^; Friend and Rainwater, 1984; Rainwater and Friend, 1987). In this theory, thermal conductivity is described according to the second thermal conductivity virial coefficient (Bλ) and depends on interatomic potential and temperature. Rainwater and Friend calculated Bλ by utilizing the Lennard-Jones potential (Rainwater and Friend, 1987). Bich and Vogel then presented correlation functions for B^*^λ (reduced second virial coefficient of thermal conductivity; Millat et al., 1996). Since the Lennard-Jones potential is a rough approximation of reality, Najafi et al. (1998) calculated the thermal conductivity of some gases at moderate density by applying a highly accurate and realistic potential known as Aziz potential (Aziz and Slaman, 1990; Aziz, 1993).

Husseinnejad and Behnejad estimated the thermal conductivity of a number of refrigerants via Bich and Vogel potential scaling parameters (Hosseinnejad and Behnejad, 2008). In 2014, Geller et al. calculated the viscosity and thermal conductivity of refrigerant mixtures using a set of models. They utilized a corresponding states method to anticipate thermal conductivity in refrigerant mixtures (Geller et al., 2014). Huber and Assael used Chapman-Enskog method to predict the transport properties of (R1234yf) and [R1234ze(E)] as refrigerant replacements (Huber and Assael, 2016). Perkins et al. represented the thermal conductivity of (R245fa) as a sum of three contributions in a range of temperatures and pressures (Perkins et al., 2016).

At densities higher than the moderate range of the RF theory, new residual correction functions were offered by Najafi et al. (1998, 2000). These functions are density-dependent. However, at higher levels of density, these methods are defeated and, thus, an appropriate method is required to solve this problem.

Recently, some nonlinear methods have been presented to classify and predict the behavior of nonlinear systems. One of the most famous approaches in these contexts is the artificial neural network (ANN). The ANN prediction method is employed in many areas including climate (Ghaderi and Darooneh, 2012; Abdellatif et al., 2015) and economy (Aydin et al., 2015) as well as in thermodynamic and thermophysics applications (Di Nicola et al., 2016; Rostamian et al., 2016).

The thermodynamic characteristics of an alternative refrigerant (R_407c_) have been examined by Sozen et al. They used an ANN to determine the specific volume, enthalpy, entropy, viscosity, and thermal conductivity of R_407c_. Promising results were obtained from the thermodynamic specifications of this refrigerant in acceptable error (Sozen et al., 2009).

The determination of thermophysical properties in the case of refrigerants was accomplished by Sencan et al. using ANN. R_413A_, R_417a_, R_422a_, R_422d_, and R_423a_ were considered while the liquid and vapor thermophysical properties of refrigerants were obtained by ANN (Sencan et al., 2011). The thermodynamic analyses of R_12_, R_22_, and R_502_ using ANNs were performed by Arcaklioglu et al. (2004). In some studies, ANN has been used for predicting the viscosity of refrigerants (Cristofoli et al., 2002; Ghaderi et al., 2013). Thermodynamic properties such as enthalpy, entropy, and specific volume of R_413a_, R_417a_, R_422d_, and R_423a_ were predicted using ANN and the adaptive neuro-fuzzy approach by Sahin et al. (2012). Furthermore, Sozen et al. utilized ANN method for estimating the thermodynamic properties of an environmentally friendly refrigerant (R_404a_) in both superheated vapor region and saturated liquid-vapor region (wet vapor) as numerical equations. In this study, the calculated thermodynamic properties had an acceptable uncertainty (Sozen et al., 2010). A three-layer feed-forward neural network was employed to predict the thermal conductivity of pure gases, and the performance of ANN was compared with those of several commonly used models. The results indicated that the commonly used regular conductivity correlations are used for a limited range of temperatures and components, while the network method can cover a wide range of temperatures and substances (Eslamloueyan and Khademi, 2009). In the case of refrigerants, the viscosity of six refrigerants was predicted by ANN and computational methods. The ANN method shows a very good accuracy at high levels of density, while computational methods are defeated in the case of density levels higher than 8 (Ghaderi et al., 2013).

In the present study, the thermal conductivity of six refrigerants containing *R*12 (dichlorodifluoromethane), *R*14 (carbontetrafluoride), *R*32 (diflouromethane), *R*143*a* (1, 1, 1trifluoroethane), *R*115 (chloropentafluoroethane), and *R*152*a* (1, 1difluoroethane) was calculated by the RF method at low and moderate levels of density. For higher ranges of density, a modified RF theory was applied which stretched beyond the validity range of the RF theory and allowed for the development of corresponding states correction functions. Residual thermal conductivity was also computed. Then, the graphs of Δλ vs. ρ were drawn. The principle of corresponding states was used to confirm the graphs. The parameters λ^*^ and ρ^*^ of refrigerants were also found.

The results of thermal conductivity calculation were compared with the existing experimental data (Keyes, 1954; Oshen et al., 1967; Rosenbaum and Thodos, 1967; Rodgers et al., 1974; Makita et al., 1981; Yata et al., 1984; Imaishi et al., 1985; Millat et al., 1988; Hahne and Song, 1989; Tanaka et al., 1991; Assael et al., 1992, 2000; Gross et al., 1992; Assael and Karagiannidis, 1993; Kim et al., 1993; Papadaki and Wakeham, 1993; Vargaftik, 1994; Gao et al., 1995; Hammerschmidt, 1995; Ro et al., 1995; Zaporozhan and Geller, 1995; Gross and Song, 1996; Krauss et al., 1996; Tsvetkov et al., 1996; McLinden et al., 2000; Le and Garrabos, 2001). After that, a feed forward ANN was used to predict the thermal conductivity of six refrigerants in the entire range of densities. The ANN training data were collected from the National Institute of Standards and Technology (NIST) (http://webbook.nist.gov/chemistry/). The NIST Chemistry Web Book has provided a great online database of chemical and physical properties for chemical species. The NIST also introduces many useful references and experiments that contain practical and experimental data.

Since there is no established method for determining the thermal conductivity of refrigerants in a wide range of densities, the ANN's efficiency was investigated. It is proposed that ANN as a nonlinear approach can successfully predict thermal conductivity. Finally, the two methods were compared. According to the results, the computational method is a reliable method only at low levels of density, while ANN is a valuable method at moderate and high levels of density where the computational method fails.

This paper is organized as follows: Computational methods for predicting thermal conductivity are described in first section. The feed-forward ANN and the learning algorithm are explained in second section. Applications of the computational method and the ANN approach for predicting thermal conductivity are stated in third and fourth sections. Finally, fifth and sixth sections are devoted to the expression of results, discussion, and conclusions.

## Computational method for predicting thermal conductivity over a wide range of densities

### Refrigerant thermal conductivity at low and moderate levels of density

Since polyatomic gas refrigerants have internal degrees of freedom, the unity between translational and internal modes has a significant influence on the thermal conductivity of these gases. The Chapman-Enskog theory is applicable only to monatomic gases with a symmetrical and spherical intermolecular potential. Therefore, the thermal conductivity of the refrigerants cannot be calculated with this theory (Felder et al., 1964).

The thermal conductivity of refrigerants in the moderate density (up to 2 mol.dm^−3^) has been calculated using the Rainwater-Friend theory. In this method, the thermal conductivity has been considered as a function of second virial coefficients (Rainwater, 1981; Rainwater and Friend, 1987):

(1) $$\begin{array}{l} \lambda =\lambda_{0}\left(1+N_{A}\sigma^{3}B_{\lambda }^{*}\rho \right) \end{array}$$

In the RF method, the Lennard-Jones potential has been used to calculate *B*_λ_ over the reduced temperature (T^*^). In this equation, λ_0_ is the thermal conductivity in zero density limits, ρ is the molar density, N_A_ is Avogadro's constant, and σ is the collision diameter. RF theory deals with realistic potentials and the reduced second thermal conductivity virial coefficient (B^*^) (Rainwater, 1981; Rainwater and Friend, 1987). B^*^ is composed of three statements:

(2) $$\begin{array}{l} B_{\lambda }^{*}=B_{\lambda }^{*\left(2\right)}+B_{\lambda }^{*\left(3\right)}+B_{\lambda }^{*\left(M-D\right)} \end{array}$$

where $B_{\lambda }^{*\left(2\right)},B_{\lambda }^{*\left(3\right)},B_{\lambda }^{*\left(M-D\right)}$ are monomer-monomer collisions, triple molecular collisions and monomer-dimer collisions, respectively. B^*^(T^*^) of a gas is dependent on the potential and the reduced temperature and is presented as:

(3) $$\begin{array}{l} B^{*}=a_{0}+\frac{a_{1}}{T^{*}} \end{array}$$

where the coefficients are:

$$\begin{array}{l} a_{0}=2.14610^{-1}\pm 7.5\times 10^{-3},a_{1}=5.359\pm 1.3\times 10^{-2} \end{array}$$

T^*^ is the reduced temperature and equals to $\frac{kT}{\varepsilon }$, where *k* is the Boltzmann constant, *T* is the absolute temperature and ϵ is the potential well depth. In this section, RF theory is developed to calculate the thermal conductivity of refrigerants. Furthermore, by applying the Mason-Monchick theory (Mason and Monchick, 1962), an appropriate correction is applied for computing the internal contribution of thermal conductivity. In the Mason-Monchick theory, the thermal conductivity of polyatomic gases is expressed as the sum of two private contributions. (Bich and Vogel, 1991):

(4) $$\begin{array}{l} \lambda =\lambda_{tr}+\lambda_{\mathrm{int}} \end{array}$$

With an acceptable approximation, these two statements of contributions are assumed to be separate. Combining the Mason-Monchick theory and Enskog hard sphere theory, the expansion of the RF theory is written as: (Vesovic and Wakeham, 1992; Heck et al., 1995)

(5) $$\begin{array}{l} \lambda_{\mathrm{int}}=\rho N_{A}DC_{\mathrm{int}} \end{array}$$

where *D* is the diffusion coefficient and *C*_*int*_ is the contribution of internal states of heat capacity. The self-diffusion coefficient is density dependent and in the Enskog theory, it is defined as (Bennett and Curtiss, 1969):

(6) $$\begin{array}{l} D=D_{0}\left(1-0.625\rho^{*}+\ldots \right) \end{array}$$

where *D*_0_ is the self-diffusion coefficient in zero density and ρ^*^ is the reduced density that is expressed as:

(7) $$\begin{array}{l} \rho^{*}=\left(\frac{2\pi }{3}\right)N_{A}\sigma^{3}\rho \end{array}$$

so the Equation (5) rearranges as:

(8) $$\begin{array}{l} \lambda_{\mathrm{int}}=\rho N_{A}D_{0}C_{\mathrm{int}}\left(1-0.625\rho^{*}+\ldots \right) \end{array}$$

by combining Equations (1, 8), the density dependence of the thermal conductivity is defined as (Mason and Monchick, 1962):

(9) $$\begin{array}{l} \lambda =\lambda_{0}\left(1+\frac{2\pi }{3}N_{A}\sigma^{3}b_{\lambda }\rho \right) \end{array}$$

where *b*_λ_ is presented as the extended RF second thermal conductivity virial coefficient for polyatomic gases. *b*_λ_ is then calculated as follows:

(10) $$\begin{array}{l} B_{\lambda }=\frac{3}{2\pi }B_{\lambda }^{*}\frac{\lambda_{0tr}}{\lambda_{0}}-0.625\left(1-\frac{\lambda_{0tr}}{\lambda_{0}}\right) \end{array}$$

and

(11) $$\begin{array}{l} \lambda_{0}=\frac{15k_{B}}{4m}\eta_{0}=\frac{15R}{4M}\eta_{0} \end{array}$$

where *k*_*B*_ is the Boltzmann constant, *M* is molecular weight and η_0_ is viscosity at zero density (Bennett and Curtiss, 1969).

### Thermal conductivity of refrigerants at higher levels of density

At the high levels of density, residual thermal conductivity is presented as Dλ. This term is a function of both temperature and density. At density levels beyond the range of the RF theory, there is a regular dependence between thermal conductivity and density which can be presented as: (Millat et al., 1996)

(12) $$\begin{array}{l} \lambda_{\left(\rho ,T\right)}=\lambda_{\left(T\right)}^{0}+D\lambda_{\left(\rho ,T\right)}+D\lambda_{c\left(\rho ,T\right)} \end{array}$$

where λ_*c*(ρ,*T*)_ is the thermal conductivity critical enhancement. The refrigerants are not considered at their critical range. Therefore, λ_*c*(ρ,*T*)_ can be ignored in the discussion of the refrigerants' behavior. It means that the residual thermal conductivity can be defined as:

(13) $$\begin{array}{l} \lambda_{\left(\rho ,T\right)}=\lambda_{\left(T\right)}^{0}+D\lambda_{\left(\rho ,T\right)} \end{array}$$

*Dλ* for aforesaid refrigerants is calculated via the following relation:

(14) $$\begin{array}{l} D\lambda =\lambda -\lambda_{0}\left(1+\frac{2\pi }{3}N_{A}\sigma^{3}b_{\lambda }\rho \right) \end{array}$$

Correction functions have been used for the corresponding states offered by Najafi et al. (2000). The curves of *Dλ* vs. ρ for six refrigerants are used to show *Dλ*/λ^*^ vs. ρ/ρ^*^:

(15) $$\begin{array}{l} \frac{D\lambda }{\lambda^{*}}=\underset{j=2,4,6,8}{\sum }d_{J}\frac{\rho^{J}}{\rho^{*}} \end{array}$$

where expansion coefficients are:

$$\begin{array}{l} d_{2}=7.257\times 10^{-2}\pm 2.01\times 10^{-3},d_{4}=1.534\times 10^{-5}\pm 9.55\times 10^{-6}\\ d_{6}=1.36\times 10^{-7}\pm 1.2\times 10^{-8},d_{8}=-4.939\times 10^{-11}\pm 4.6\times 10^{-12} \end{array}$$

Thermal conductivity for each of the refrigerants has been obtained by a simple equation:

(16) $$\begin{array}{l} \lambda =\lambda^{0}\left(1+b_{\lambda }\rho^{*}\right)+D\lambda \end{array}$$

### ANN, MLPs, and back-propagation algorithm

ANNs are models of the cognitive process of the brain with an incredible potential for advancing the types of problems solved by computers (Steeb, 2005). The neuron is the basic processor in these models. Each neuron receives several inputs from other neurons. In 1958, perceptron was suggested as the simplest model of computational neuron by Rosenblatt. Perceptron is used for classifying linearly separable patterns. The network which consists of a set of perceptrons in a layer-by-layer structure is known as Multi-Layer Perceptrons (MLPs). In layered architectures, the first-layer perceptrons (input layer) are only connected to the second-layer units (first hidden layer) and so on (Ghaderi and Darooneh, 2012). The input signal propagates through the network in a forward direction and there is no recurrent connection in these networks. Consequently, these networks are called multi-layer feedforward ANNs. The model of each neuron in the MLP includes a nonlinear activation function. Therefore, the output of each perceptron is:

(17) $$\begin{array}{l} y_{k}=sig\left(\overset{n}{\underset{i=0}{\sum }}w_{ik}x_{i}\right) \end{array}$$

where *w*_*ik*_ is synaptic weight of the neuron *k, xi* is input to the each neuron and *n* is the number of inputs. *sig* is a nonlinear sigmoid function. The output of these functions is in the range of −1 to +1 or 0 to +1.

In a multilayer feed-forward network, several learning algorithms can be applied for network training. The back-propagation algorithm is a powerful method which is operated based on the gradient descent rule in the weight space. Three stages are implemented in this algorithm: (1) the feed-forward of input signals, (2) the calculations of the associated error and back-propagations of the error signal, and (3) the improvement of synaptic weights [79]. The error signal is calculated by subtracting the desired output from the network output. Using the error signal, the instantaneous value of error energy for neuron *j* is indicated by:

(18) $$\begin{array}{l} E\left(n\right)=\frac{1}{2}\underset{1\to n}{\sum }e_{j}^{2}\left(n\right) \end{array}$$

where *n* is the number of neurons in the output layer. The associated error in the back-propagation algorithm is calculated using average squared error energy:

(19) $$\begin{array}{l} E_{av}=\frac{1}{N}\overset{N}{\underset{n=1}{\sum }}E\left(n\right) \end{array}$$

where *E*(*n*) is error energy for neuron *j*.

### Application of computational method for predicting of thermal conductivity

The six refrigerants including R_12_, R_14_, R_32_, R_115_, R_143_, and R_152_ are polyatomic. As a result, available experimental data should be used to obtain λ_0_ (thermal conductivity in the zero density). Rainwater-Friend theory was employed for moderate density range (Equation 1). In addition, the value of η_0_ (Equation 11) was calculated for these six refrigerants in Ghaderi et al. (2013).

**Figure 1:**
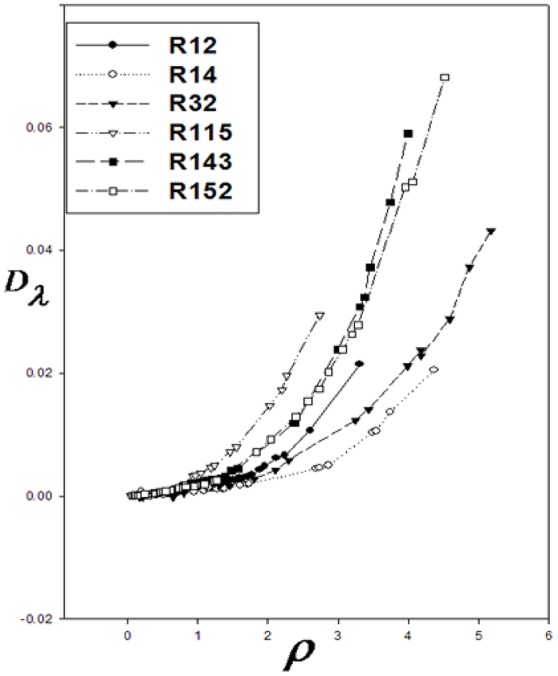
*D*_λ_ vs. ρ for six refrigerants.

**Figure 2:**
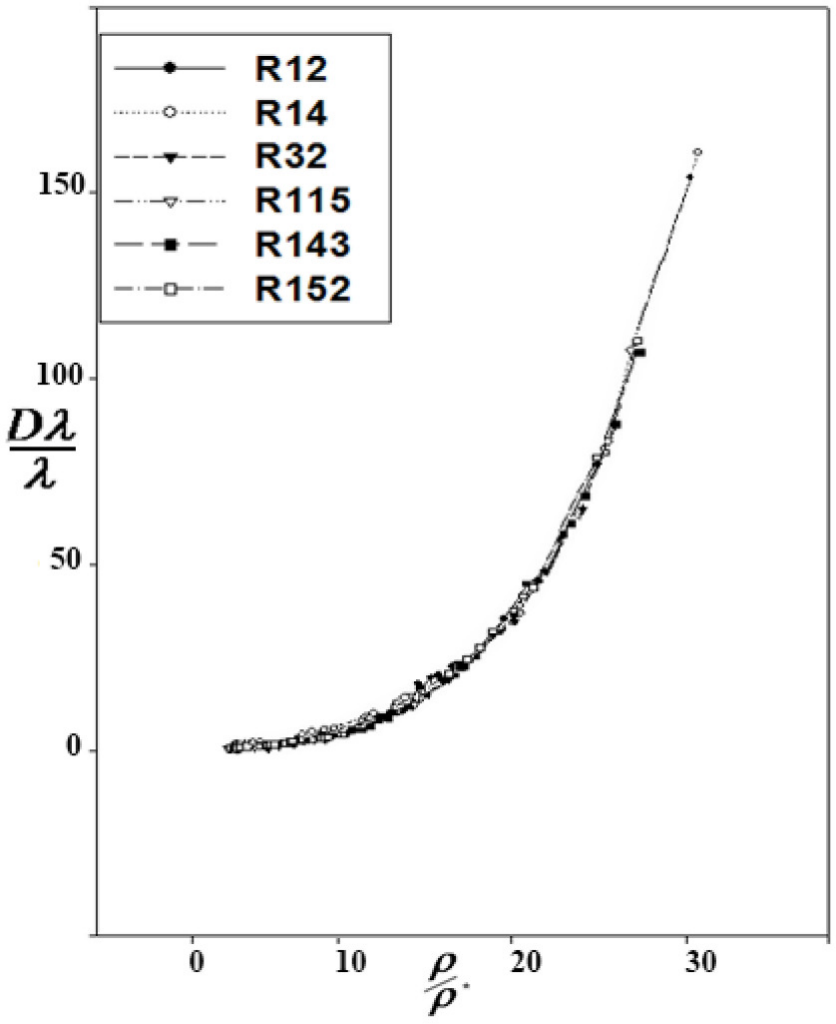
Reduced *D*_λ_ vs. reduced ρ according to corresponding states principle.

At the high densities, D_λ_ is dependent on density. Therefore, different isotherms of the residual thermal conductivity will confirm on a single curve. D_λ_ was calculated using Equation 14, and D_λ_ vs. ρ were drawn for the aforementioned refrigerants (Figure 1). Using the Sigma-Plot software, adjust quantities λ^*^ and ρ^*^ were found. The corresponding states principle was applicable for the thermal conductivity as a transport property. It means that the six curves were fitted on, as shown in Figure 2. The values obtained by the computational method are compared with the experimental values in Table 1. The calculated values of the noted refrigerants are in good agreement with the experimental values. Adjust quantities and predicted values for thermal conductivity are compared with the experimental data for six refrigerants in Table 1. The results are in agreement with the experimental data outside the critical area.

**Table 1:** Values obtained by computational method were compared with the experimental data of λ for 6 refrigerants in low and low-moderate density.

**Fluid**	***ρ_*min*_–ρ_*max*_***	***T_*min*_–T_*max*_***	λ*	ρ*	**Av.(Max)%Δ**	**No. points**
*R*_12_	0.24–3.3	350–500	1.2 × 10^−4^	9.8 × 10^−2^	0.9 (2.9)	31
*R*_14_	0.1–4.3	200–600	1.4 × 10^−4^	0.1298	1.3 (3.3)	41
*R*_32_	0.17–5.1	200–600	5.7 × 10^−4^	0.2041	1.3 (2.5)	19
*R*_115_	0.04–2.7	250–500	1.2 × 10^−3^	0.1629	1.1 (2.6)	35
*R*_143_	0.7–4.0	342–600	4.7 × 10^−4^	0.135	1.1 (2.9)	25
*R*_152_	0.7–4.5	275–500	5.9 × 10^−4^	0.1501	0.8 (3.5)	41

### Predicting thermal conductivity by ANN at low, moderate, and high levels of density

A three-layer feed-forward ANN was constructed using MATLAB neural network toolbox. Three neurons were considered in the input layer and one neuron in the output layer. The three input variables were temperature, density, and pressure, while the output variable was thermal conductivity. These variables proved to be effective parameters on the thermal conductivity value.

**Figure 3:**
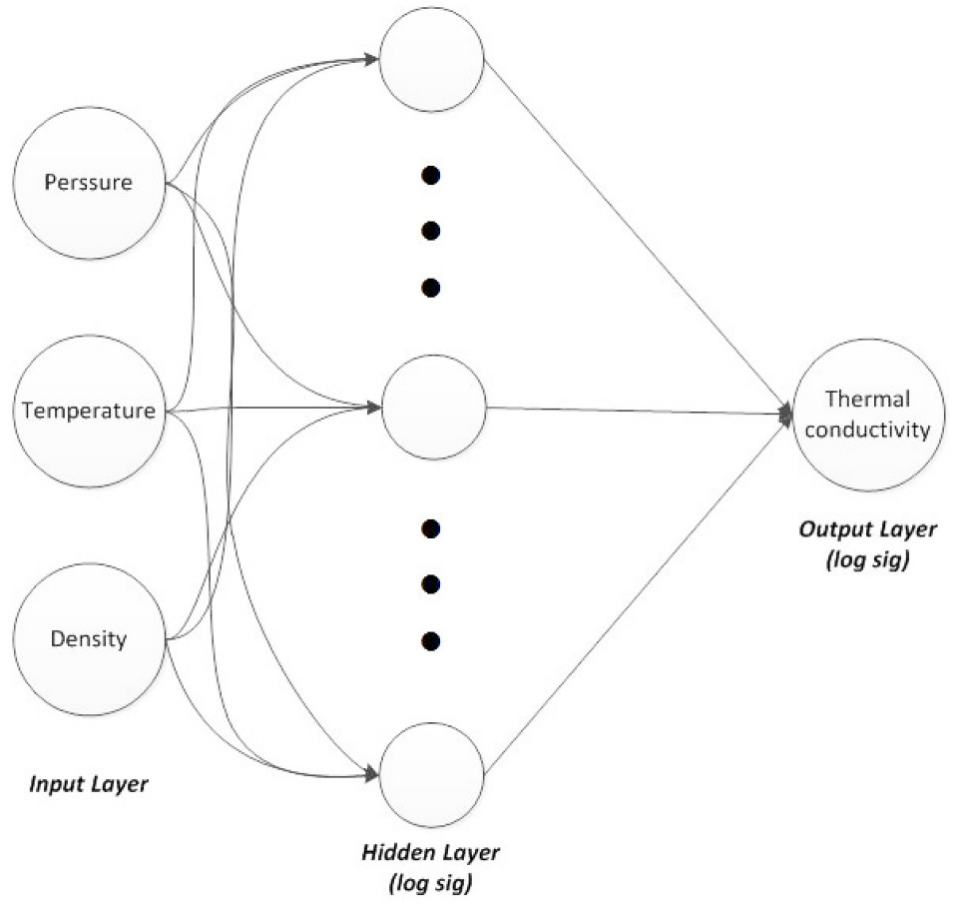
Three-layers neural network, three neurons in input, 10, 20, and 30 nodes in hidden layer and one node in output.

The ANN structure is illustrated in Figure 3. Resilient back-propagation algorithm was used as the training algorithm and a nonlinear sigmoid function (logsig), which showed better results than the other sigmoid function (tansig), was employed as the activation function in the hidden and output layers. The logsig function is defined as:

(20) $$\begin{array}{l} \log sig\left(n\right)=\frac{1}{1+e^{-n}} \end{array}$$

where *n* is the input value. Additional data about this network are presented in Table 2. NIST data were used as the training data, while the testing data were obtained from experimental references. The number of training and testing patterns of the six refrigerants is indicated in Table 3.

**Table 2:** Additional data about ANN structure.

**Learning rate**	**Act. Func. (hidden)**	**Act. Func. (output)**	**Training algorithm**	**Epochs**
0.05	logsig	Logsig	trainrp	30,000

**Table 3:** Number of training and testing patterns for ANN training.

**Fluid**	**No. of training patterns**	**No. of testing patterns**
*R*_12_	393	65
*R*_14_	347	57
*R*_32_	350	58
*R*_115_	350	58
*R*_143_	377	62
*R*_152_	391	65

**Figure 4:**
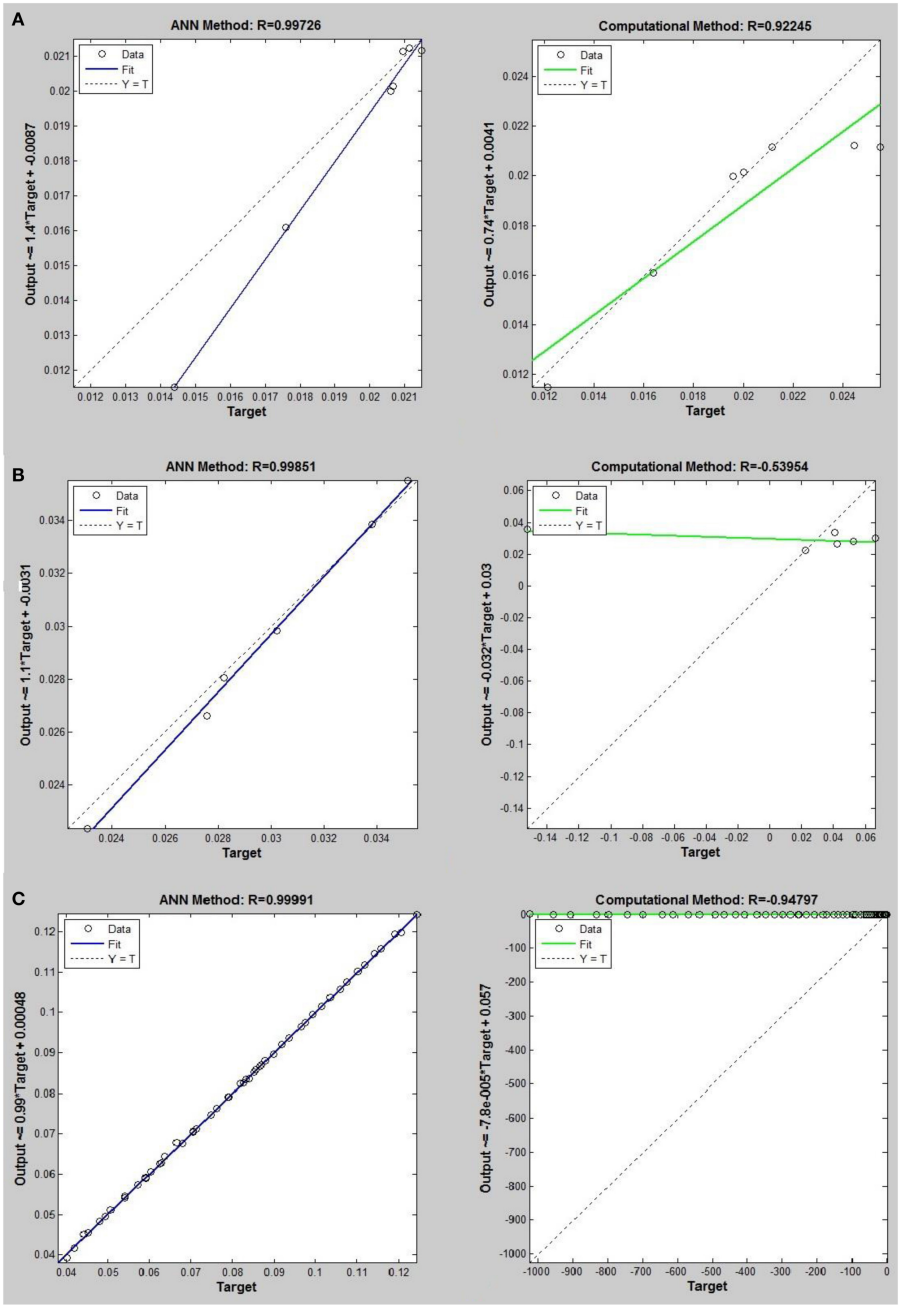
Thermal conductivity regression. Comparison between computational and ANN Methods for *R*12. **(A)** Comparition in low density (ρ < 2). **(B)** Comparison in moderate density (2 < ρ < 6). **(C)** Comparison in high density (ρ > 6).

**Figure 5:**
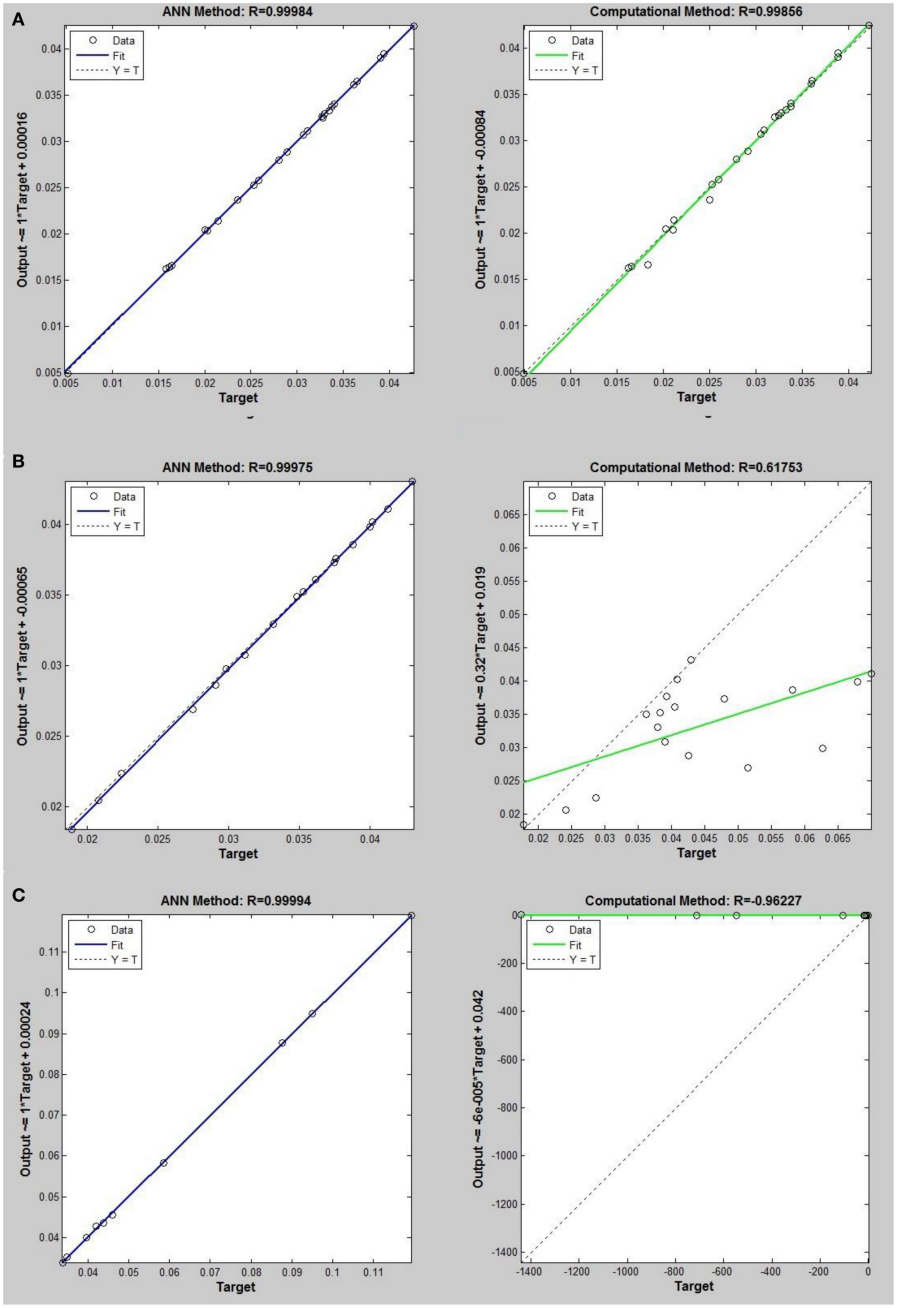
Thermal conductivity regression. Comparison between computational and ANN Methods for *R*14. **(A)** Comparison in low density (ρ < 2). **(B)** Comparison in moderate density (2 < ρ < 6). **(C)** Comparison in high density (ρ > 6).

**Figure 6:**
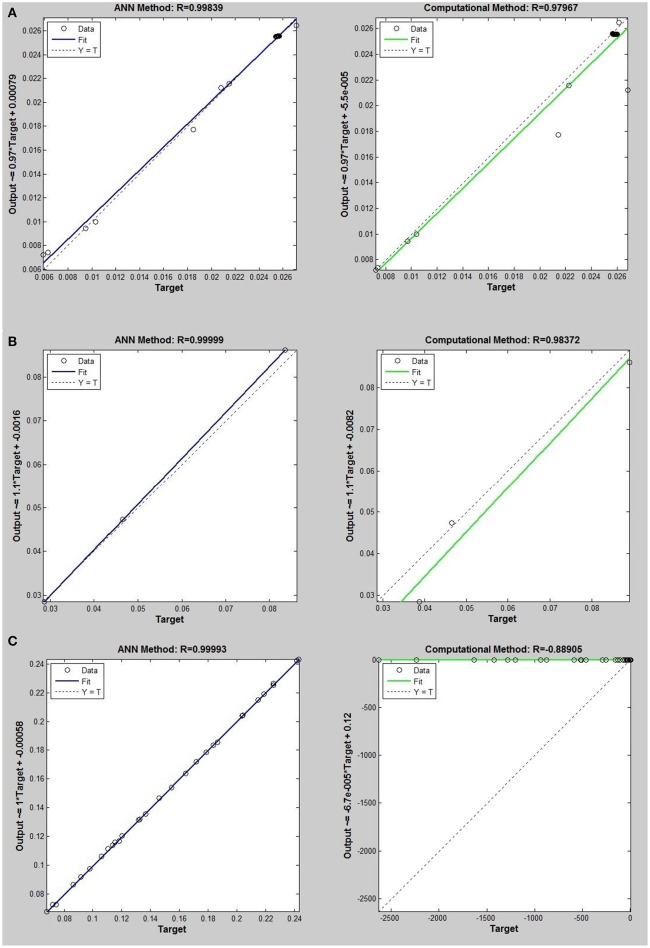
Thermal conductivity regression. Comparison between computational and ANN Methods for *R*32. **(A)** Comparison in low density (ρ < 2). **(B)** Comparison in moderate density (2 < ρ < 6). **(C)** Comparison in high density (ρ > 6).

**Figure 7:**
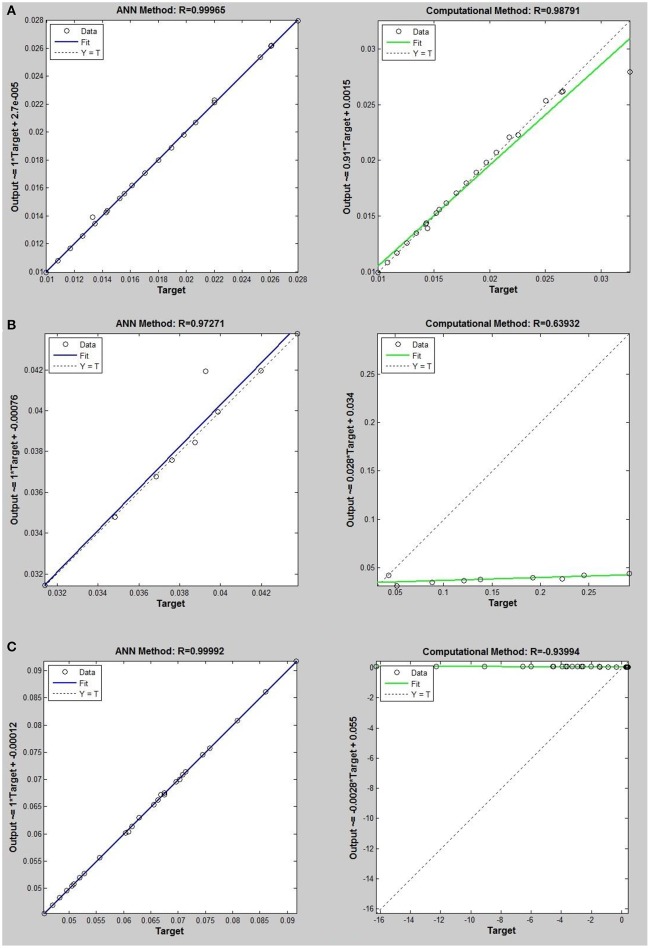
Thermal conductivity regression. Comparison between computational and ANN Methods for *R*115. **(A)** Comparison in low density (ρ < 2). **(B)** Comparison in moderate density (2 < ρ < 6). **(C)** Comparison in high density (ρ > 6).

**Figure 8:**
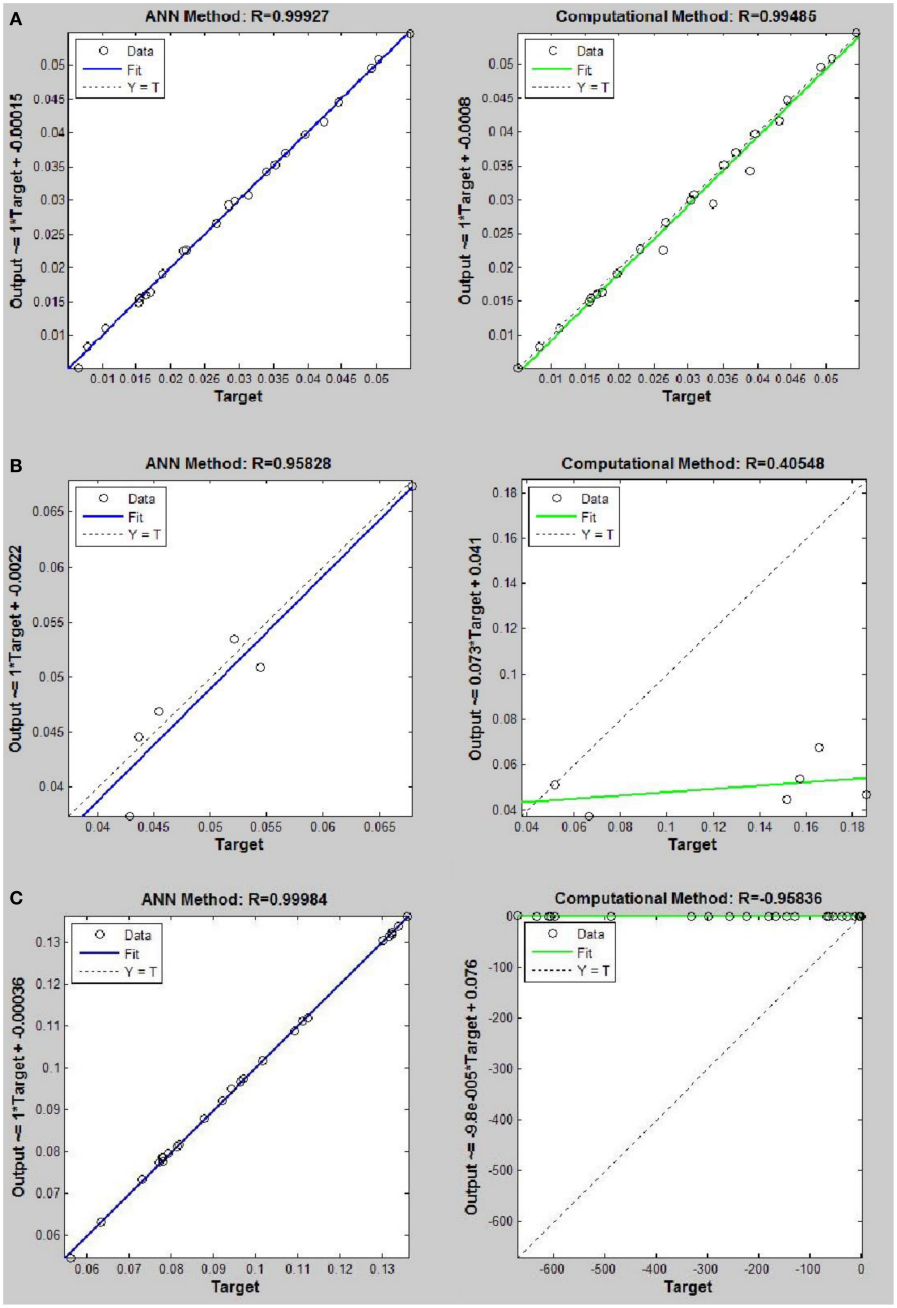
Thermal conductivity regression. Comparison between computational and ANN Methods for *R*143. **(A)** Comparison in low density (ρ < 2). **(B)** Comparison in moderate density (2 < ρ < 6). **(C)** Comparison in high density (ρ > 6).

**Figure 9:**
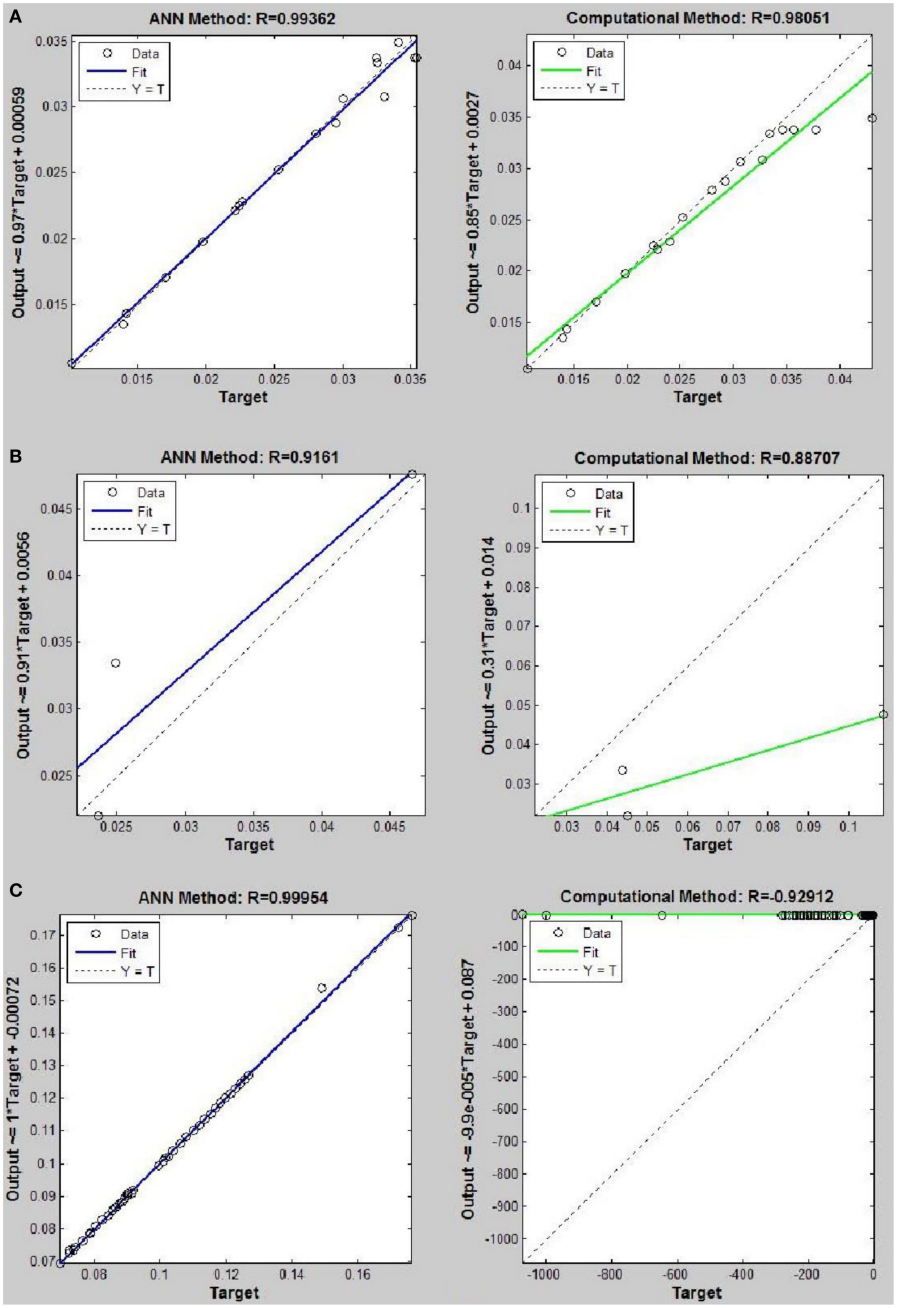
Thermal conductivity regression. Comparison between computational and ANN Methods for *R*152. **(A)** Comparison in low density (ρ < 2). **(B)** Comparison in moderate density (2 < ρ < 6). **(C)** Comparison in high density (ρ > 6).

Networks with 10, 20, and 30 neurons in the hidden layer were tested and the performances of each network in low, mid, and high densities are represented in Tables 4–6, respectively. The efficiency of the ANN method for predicting the thermal conductivity of the six refrigerants and a comparison of this method with computational method are depicted in Figures 4–9 and Tables 4–6.

**Table 4:** The R values of computational method and ANN with 10, 20, and 30 hidden neurons, for thermal conductivity prediction in low density.

**Fluid**	**ANN 10 nodes**	**ANN 20 nodes**	**ANN 30 nodes**	**Computational method**	**Density range**	**No. of patterns**
*R*_12_	**0.99726**	0.98225	0.99494	0.92245	0.30 < ρ < 1.90	6
*R*_14_	0.99909	0.99893	**0.99984**	0.99856	0.05 < ρ < 1.73	25
*R*_32_	**0.99839**	0.99226	0.99837	0.97967	0.00 < ρ < 1.72	21
*R*_115_	0.99482	0.99632	**0.99965**	0.98791	0.05 < ρ < 1.58	22
*R*_143_	0.99964	0.99927	**0.99980**	0.99485	0.00 < ρ < 1.72	23
*R*_152_	0.98677	**0.99362**	0.99064	0.98051	0.02 < ρ < 1.85	17

*The best accuracy of prediction has been indicated in bold type*.

**Table 5:** The R values of computational method and ANN with 10, 20, and 30 hidden neurons, for thermal conductivity prediction in mid density.

**Fluid**	**ANN 10 nodes**	**ANN 20 nodes**	**ANN 30 nodes**	**Computational method**	**Density range**	**No. of Patterns**
*R*_12_	0.99692	0.97797	**0.99851**	−0.53954	2.34 < ρ < 5.66	6
*R*_14_	0.99790	**0.99975**	0.99955	0.61753	2.27 < ρ < 5.84	18
*R*_32_	0.99944	**0.99999**	0.99976	0.98372	2.56 < ρ < 5.81	3
*R*_115_	0.55564	0.60352	**0.97271**	0.63932	2.72 < ρ < 5.83	9
*R*_143_	0.95954	0.95828	**0.99542**	0.40548	3.35 < ρ < 5.63	3
*R*_152_	0.85657	**0.91610**	0.88369	0.88707	2.09 < ρ < 4.22	3

*The best accuracy of prediction has been indicated in bold type*.

**Table 6:** The *R*-values of computational method and ANN with 10, 20, and 30 hidden neurons, for thermal conductivity prediction in high density.

**Fluid**	**ANN 10 nodes**	**ANN 20 nodes**	**ANN 30 nodes**	**Computational method**	**Density range**	**No. of patterns**
*R*_12_	0.99977	0.99965	**0.99991**	−0.94797	6.38 < ρ < 14.20	52
*R*_14_	0.99304	**0.99994**	0.99956	−0.96277	7.42 < ρ < 19.72	11
*R*_32_	0.99967	0.99819	**0.99993**	−0.88905	9.22 < ρ < 27.41	33
*R*_115_	0.99745	0.99836	**0.99992**	−0.93994	6.13 < ρ < 11.30	27
*R*_143_	0.99978	**0.99984**	**0.99984**	−0.95836	7.55 < ρ < 15.77	25
*R*_152_	0.99856	0.99112	**0.99954**	−0.92912	9.24 < ρ < 18.06	44

*The best accuracy of prediction has been indicated in bold type*.

## Results and discussion

In the case of calculating the thermal conductivity of the six refrigerants, the efficiency of the two methods (ANN and the computational approach) was compared. The RF theory is a statistical method for predicting the thermal conductivity of refrigerants in low densities (Rainwater, 1981; Rainwater and Friend, 1987). Nevertheless, this model fails at higher levels of density. To predict thermal conductivity at higher densities, the RF theory can be used (Najafi et al., 2000).

On the other hand, the ANN approach is a nonlinear method which can be applied for predicting thermal conductivity in all ranges of density (Sencan et al., 2011; Ghaderi et al., 2013). The performances of the ANN approach and the computational method were compared at low, mid, and high levels of density, with the results presented in Tables 4–**6**, respectively. At the low densities (<2), the two methods were reliable. However, the ANN approach had better results (Table 4). These results show that, in the case of *R*14, *R*32, *R*115, *R*143, *R*153, all of the three ANNs represented slightly better predictions than the computational method. In the moderate range of density, (Tables 1, 5 and Figures 4–9), the computational method could be used only for R_32_ and was defeated in other cases. In another study, however, the computational method showed reliable results in this range of density for predicting the viscosity of the same fluids (Ghaderi et al., 2013). Furthermore, the thermal conductivity of the six refrigerants was precisely predicted by ANN in the mid-density range.

In the high range of density, the computational method was invalid for the six refrigerants, while the ANN approach proved to be a reliable method for predicting thermal conductivity at density levels up to 27. These results are consistent with the results of another study on viscosity prediction (Ghaderi et al., 2013).

The number of hidden neurons in the ANN structure is selected as 10, 20, and 30. Based on the results, a network with 30 hidden neurons has the best prediction at higher levels of density. On the other hand, the results of the computational method indicate that the regular dependence between thermal conductivity and density at higher densities is eliminated. It can develop a nonlinear problem. Therefore more nodes in hidden layer are needed to solve the nonlinearity problem.

## Conclusion

The ANN approach proved to be a reliable method for predicting thermal conductivity in all ranges of density, while the computational method was restricted to low densities (Figures 4–9 and Tables 1, 4–6). In the low levels of density (ρ < 2), the computational method based on RF theory was usable (*R* > 0.92) (Tables 1, 4 and Figures 4–9). However, in the moderate and high levels of density (ρ > 2), this model often failed.

On the other hand, the results showed that, in the case of R32, this model succeeded at moderate levels of density (*R* = 0.98372). Thus, according to the results, we concluded that the computational method is an accurate model at low levels of density for predicting thermal conductivity prediction. Nevertheless, since the accuracy of computational method based on RF theory is reduced at higher levels of density, the ANN with MLPs as a nonlinear method is proposed for solving this problem. There is a nonlinear relationship between the thermal conductivity and other thermodynamical properties such as, pressure and density. As a result, analytical approaches are unable to predict thermal conductivity in a wide range of densities. Instead, a nonlinear approach such as, ANN is a valuable method for this purpose.

Other thermodynamic properties may be predicted by the ANN approach and the accuracy of predictions can be compared with those of computational methods. Moreover, other nonlinear methods may be used for prediction in this context.

## Author contributions

FG worked on theoretical concept of chemical aspects and computational method and wrote a considerable volume of manuscript. AG worked on artificial neural network model, compared results of two methods, and wrote a part of manuscript. NG wrote a part of manuscript and helped in data analyzing. BN conducted group in all procedures.

### Conflict of interest statement

The authors declare that the research was conducted in the absence of any commercial or financial relationships that could be construed as a potential conflict of interest.
